# Neutrophil-to-lymphocyte ratio in type 2 diabetes patients combined with Lower Extremity Peripheral Artery Disease

**DOI:** 10.3389/fendo.2024.1434580

**Published:** 2024-08-30

**Authors:** Li Li, Mengjie Wang, Ting Jia, Xiaowan Jiang, Fan Yang, Zhongjing Wang, Xuyan Zhang

**Affiliations:** ^1^ Department of Endocrinology, The Central Hospital of Wuhan, Tongji Medical College, Huazhong University of Science and Technology, Wuhan, China; ^2^ Key Laboratory for Molecular Diagnosis of Hubei Province, The Central Hospital of Wuhan, Tongji Medical College, Huazhong University of Science and Technology, Wuhan, China; ^3^ Hubei Regenerative Medicine Clinical Research Center, Wuhan, China; ^4^ Wuhan Diabetes Clinical Research Center, Wuhan, China

**Keywords:** type 2 diabetes mellitus, lower extremity peripheral artery disease, neutrophil-to-lymphocyte ratio, angiography, stenosis

## Abstract

**Objective:**

This study explored the utility of NLR (neutrophil-to-lymphocyte ratio) as a marker to predict Lower Extremity Peripheral Artery Disease (PAD) in the Chinese population, as well as to assess its consistency and diagnostic value with digital subtraction angiography.

**Methods:**

Patients were distributed into three groups according to the angiography in lower limb arterial: group L1, plaque with no stenosis; group L2, plaque with luminal stenosis and group L3, total vascular occlusion. Changes in the neutrophil-to-lymphocyte ratio were documented and compared among groups.

**Results:**

Compared to group L1, NLR was significantly increased in L2 (1.76 *vs* 2.35, *p*=0.037) and L3 (1.76 *vs* 3.60, *p<*0.001), with a gradual decrease in ABI (Ankle-Brachial Index, 1.11 *vs* 1.02 *vs* 0.94, *p<*0.001). Those older patients with higher prevalence of hypertension (*p*=0.002), obesity (*p*=0.032), or reduced high-density lipoprotein cholesterol (*p*=0.020) were more likely to develop PAD; higher glycosylated hemoglobin (*p*=0.045), low-density lipoprotein cholesterol (*p*=0.006), and systolic blood pressure (*p<*0.001) levels led to a greater tendency to suffer stenosis or even occlusion; the probability of severe stenosis (>70%) increased to 2.075 times for every 1 increase in NLR, while it was 46.8% for every 0.1 increase in ABI. The optimal NLR cut-off value to predict severe stenosis in PAD was 2.73. Receiver operating characteristic curve analysis of the inflammatory biomarkers and severe stenosis prediction displayed an area under the curve of 0.81.

**Conclusion:**

NLR could serve as a new noninvasive and accurate marker in predicting PAD.

## 1 Introduction

Diabetes mellitus (DM) is a disease characterized by the poor control of blood glucose, the prevalence is predicted to be 12.2% by 2045 (1). Patients with DM are at high risk for various cardiovascular diseases (CVD), e.g., coronary artery disease (CAD), stroke, and peripheral artery disease (PAD), which are the leading causes of DM-related mortality and morbidity. PAD is characterized by atherosclerotic stenosis or obstruction of the limb arteries, it can occur in the upper limbs, but more often in the lower limbs (2). From the epidemiological perspective, PAD occurs in patients with DM at a two- to four-fold higher incidence than in whom without DM (3), and the disease progression is more severe in the diabetic population (4). PAD affects 20–28% of the diabetic population, and up to 50% of patients with diabetic foot disease (DFD) (2). In China, the occurrence of peripheral artery disease in type 2 diabetic cases is reported to be 55.3-65% in different studies (5, 6). As claimed by the China DIA-LEAD epidemiological investigations, the currency of type 2 diabetes mellitus combined with lower extremity PAD was 21.2% in patients over 50 years old (7). What is more, PAD can result in a threat to the quality of life and survival period in diabetic individuals (8–10). In the United States, 4.6% of diabetic patients develop lower extremity chronic ulcers (at least 6 weeks) or amputations below the knee (of at least one toe) (11). So, early PAD diagnosis in diabetic patients is particularly important for preventing complications, such as ulcers or gangrene, and for reducing major adverse limb events, adverse cardiovascular events, and mortality. However, PAD diagnosis in diabetic patients is often made difficult by the characteristics of the diseases. Characteristic PAD symptoms are frequently absent in diabetic patients. Often, patients do not report claudication due to a lack of physical activity, and do not refer pain due to concomitant neuropathy (12). This implies that PAD diagnosis is made already at the most advanced stages of the disease, often when patients already present DFD. Currently, diagnosis of lower extremity arterial stenosis is dependent on lower extremity arteriography (Digital Subtraction Angiography, DSA) as an assessment (13), its great advantage is that it is diagnostic and can be interventional at the same time (14). However, due to its high technical difficulty, invasiveness, high cost, and possible postoperative complications such as hematoma, bruising, and even contrast nephropathy, the widespread implementation of DSA has been limited; meanwhile, the traditional Doppler ultrasound diagnosis, although convenient and economical, is still difficult to accurately screen patients with lower extremity occlusion at an early stage (15), it is operator-dependent particularly in the case of calcification or structures close to bone or gas-filled cavities (16). ABI is currently a valid measurement for the diagnosis of PAD, but it is usually higher than actual in diabetic foot ulcers because of its association with vascular calcification and impaired elasticity (17). Therefore, there is an urgent need for a screening tool that can accurately and early detect diabetic lower limb vascular lesions but is also convenient, cost-effective, and easy to perform widely for early detection and intervention.

Studies have shown that many risk factors such as hyperglycemia, hyperlipidemia, and hypertension can lead to atherosclerosis. From a pathophysiological perspective, various inflammatory cytokines including chemokines, adipokines, adhesion molecules, and cytokines may contribute to the development of peripheral artery disease in the lower limbs in diabetes. In the early stages, oxidative stress accompanied by inflammatory responses occurs after the elevation of blood glucose, massive neutrophil infiltration mediates non-specific inflammatory response (18, 19), causing vascular endothelial damage, followed by a buildup of plaque in the inner arterial wall (20), the accumulation of neutrophil protease further drives plaque instability, even plaque hemorrhage and plaque detachment (21). At the same time, damaged vascular endothelial cells express chemokines and adhesion molecules can recruit lymphocytes to infiltrate the endothelium (22): lymphocytes is an immune system regulation pathway, by reducing the number of CD8^+^ T lymphocytes to inhibit the anti-inflammatory environment, maintaining low chronic inflammation, long-term inflammation leads to the proliferation of vascular smooth muscle, microangiogenesis, subsequent arterial lesions and plaque formation (23). To make matters worse, decreased neutrophil apoptosis and the increase of lymphocyte apoptosis lead to abnormal cell ratio mediating insulin resistance and insulin secretion dysfunction, accelerating the production of more active oxidants, leading to permeability and dysfunction of vascular endothelial cells, decreased nitric oxide and capillary expansion, eventually reducing microcirculation to peripheral tissues (24), followed by atherosclerotic plaques and varying degrees of stenosis even up to occlusion (25), which we called PAD.

Several studies have shown that PAD is associated with pro-inflammatory cytokines, such as interleukin-6, interleukin-1, and tumor necrosis factor (26, 27). However, the detection of the inflammatory markers mentioned above is not used in common work because of the payments and technical hardships. The blood-routine test is a sensitive and cost-effective detection that can be done easily in the laboratory, the neutrophil-to-lymphocyte ratio (NLR) is currently a novel predictor of inflammation (28), it has been monitored in other diseases such as coronary heart disease (29), tumors (30, 31), thyroid disease (32). Studies have shown that NLR is a risk factor for elevated blood glucose, positively correlates with HbA1c levels in patients with T2DM (33, 34), and is involved in the development of diabetes-related complications including diabetic nephropathy (35) or diabetic retinopathy (36), but the role and function of NLR in diabetes mellitus combined with PAD has not been studied. Based on the description of the changes and roles of neutrophils and lymphocytes in atherosclerosis in the paragraph above, therefore, this study proposed to assess the change of NLR in diabetic PAD and whether or not NLR can serve as a better predictor.

## 2 Materials and methods

### 2.1 Study design and participants

Between January 2021 and June 2023, 194 diabetic patients with PAD (age 18-75 years) were enrolled. Inclusion criteria: (1) All patients were diagnosed type 2 diabetes mellitus according to the 2021 American Diabetes Association (ADA) criteria (37); (2) All patients were accompanied by different degrees of distal lower limb ischemia symptoms, manifested as intermittent limp and rest pain, or some patients were asymptomatic, manifested only by low skin temperature of the extremities, and diminished or absent dorsal pedis artery or posterior tibial artery pulsations found during physical examination (The Leriche Fontaine and Rutherford classifications are based on clinical symptomatology, **Table 1**.); (3) All patients underwent lower extremity arteriography and had varying degrees of vascular plaque or stenosis. Exclusion criteria: (1) ketosis, diabetic nonketotic hyperosmolar coma, acute cardiovascular and cerebrovascular disease, and other states of stress; (2) acute infections in the combined lower limbs or elsewhere; (3) special types of diabetes mellitus; (4) acute or chronic renal insufficiency (eGFR <60ml/min/1.73m^2^) or coagulation disorders; and (5) hepatitis, cirrhosis, tuberculosis, tumors, or other immune-deficiency diseases, or those who were receiving hormone therapy.

**Table 1:** Demographic and laboratory characteristics of the study population.

Characteristic	Group 1 (no stenosis) N=56	Group 2 (stenosis) N=58	Group 3 (occlusion) N=80	*p*-value
Male/Female (N)	32/24	36/22	48/32	0.93
Age (year)	58.61 ± 7.38^a,c^	64.66 ± 8.94^a^	68.33 ± 9.52^c^	***<0.001***
Duration (year)	7.5 (4.0-10.0)^a,c^	10 (9.0-15.0)^a^	10.0 (8.0-16.5)^c^	***0.01***
Smoking (Yes/No)	16/40	26/32	44/36	0.338
BMI (Kg/m^2^ )	22.2 (20.0-23.5)^a,c^	23.2 (21.35-25.5)^a^	25.6 (22.2-27.9)^c^	***0.032***
Hypertension (Yes/No)	20/36^a,c^	38/20^a^	62/18^c^	***0.002***
SBP (mmHg)	127.5 (120.0-130.0)^c^	125.0 (120.0- 150.0)^b^	150.0 (136.0-159)^b,c^	***<0.001***
DBP (mmHg)	80.0 (70.0-85.2)	77.0 (70.0 - 85.5)	74.0 (70.0-80.0)	0.601
HbA1c (%) (mmol/mol)^d^	8.6 (7.20-10.15)^c^ 70.49 (55.19-87.43)	8.9 (7.60- 10.25) 73.77 (59.56-88.52)	9.65 (8.6-11.2)^c^ 81.97 (70.49-98.91)	***0.045***
TG (mmol/L)	4.26 ± 1.07	4.46 ± 1.44	3.93 ± 1.43	0.251
TC (mmol/L)	3.58 (2.30-4.92)	3.22 (2.15-4.20)	2.42 (1.29-3.81)	0.090
HDL (mmol/L)	1.15 (1.01-1.33)^a,c^	1.11 (1.00-1.41)^a^	1.05 (0.87-1.14)^c^	***0.020***
LDL-c (mmol/L)	2.31 (1.59-2.57)^c^	2.52 (1.84-2.93)^b^	2.88 (2.29-3.60)^b,c^	***0.006***
eGFR (ml/min/1.73m^2^)	96.17 ± 10.92	92.74 ± 8.54	84.63 ± 7.62	**0.072**
WBC (*10^9^ /L)	5.82 (4.97-6.77)	5.80 (4.98-7.42)	6.88 (5.35-8.76)	0.080
Neutrophil count (*10^9^/L)	3.30 (2.75-3.98)^c^	3.51 (2.87-5.25)	4.30 (3.08~6.16)^c^	***0.012***
Lymphocyte count (*10^9^/L)	1.96 (1.58-2.35)^c^	1.68 (1.22-2.14)	1.38 (1.16-2.07)^c^	***0.012***
CRP( (mg/L)	1.69 (0.85-3.42)^c^	1.97 (1.02-4.09)	2.26 (1.56-4.97)^c^	***0.036***
Rutherford classification, % (n/N)				
1	57.14 (32/56)	44.83 (26/58)	36.25 (29/80)	
2	37.50 (21/56)	42.38 (24/58)	35.00 (28/80)	
3	5.36 (3/56)	3.45 (2/58)	17.50 (14/80)	
4	(0/56)	3.45 (2/58)	11.25 (9/80)	
Leriche–Fontaine classification, % (n/N)				
IIa	57.14 (32/56)	44.83 (26/58)	36.25 (29/80)	
IIb	42.86 (24/56)	51.72 (30/58)	52.50 (42/80)	
III	0 (0/56)	3.45 (2/58)	11.25 (9/80)	
Vessel lesions, % (n/N)				
Above The Knee	/	46.55 (27/58)	13.75 (11/80)	
Below The Knee	/	53.45 (31/58)	86.25 (69/80)	
NLR	1.76 (1.28-2.23)^a,c^	2.35 (1.71-3.24)^a,b^	3.60 (2.66-6.02)^b,c^	***0.001***
ABI	1.11 ± 0.14^a,c^	1.02 ± 0.12^a,b^	0.94 ± 0.13^b,c^	***<0.001***

Data shown as mean ± SD for continuous variables, and median (interquartile range) for skewed data.

Bold values indicate statistical significance. ^a^Comparison of group 1 and group 2; ^b^Comparison of group 2 and group 3; ^c^Comparison of group 1 and group 3; ^d,^ HbA1c values were calculated as mmol/mol, the NGSP converter is available online (http://www.ngsp.org/convert1.asp).

BMI, body mass index; SBP, systolic blood pressure; DBP, diastolic blood pressure; HbA1c, Hemoglobin A1c; TG, triglycerides; TC, total cholesterol; HDL-C, high-density lipoprotein cholesterol; LDL-c, low-density lipoprotein cholesterol; WBC, white blood cell; eGFR, Estimated glomerular filtration rate; CRP, C-reactive protein; NLR, the neutrophil-to-lymphocyte ratio; ABI, ankle-brachial index.

The study was a retrospective analysis, data were obtained from patient records file regarding age, sex, duration of diabetes, height, weight, blood pressure, smoking history, Body Mass Index (BMI) =weight/(height squared) (international unit kg/m^2^) and other necessary information.

This study protocol conforms to the ethical guidelines of the 1975 Declaration of Helsinki, and the research protocol was priorly approved by the Ethics Committee of Wuhan Central Hospital (Ethical No. WHZXKYL2023-186). Written informed consent was obtained from all the patient to process their personal data for scientific research purpose.

### 2.2 Laboratory measurements

Blood samples (fasted for more than 8 hours) were drawn from all patients on the day after admission. A routine blood test was measured using mindary BC-6800 (flow cytometric analysis), assessment of NLR dividing the absolute neutrophil count on the absolute lymphocyte count, lipid profile was quantified using Beckman Coulter AU5800 (enzyme electrode method), and HbA1c was measured using D-10 Glycated Hemoglobin Meter (microcolumn method), serum CRP (C-reactive protein) has been determined using turbidimetry (Beckman Coulter, Brea, USA). ABI was evaluated by VBP-9 atherosclerosis detector.

### 2.3 Digital subtraction angiography

DSA was carried out by Advantx LCV digital subtraction angiography equipment of GE, and the right femoral artery was punctured by using a 5F-6F catheter, which was sent to the bifurcation of the abdominal aorta under fluoroscopy, and the lower limb vascular visualization was observed. Patients were sorted into three groups: group L1, plaque with no stenosis; group L2, plaque with luminal stenosis (L2a: stenosis<70%; L2b: stenosis at 70%-99%) and group L3, total vascular occlusion in lower limb arterial.

### 2.4 Statistical analysis

Statistical Package for Social Science (SPSS, version 27.0; SPSS, Inc., Chicago, USA) was applied for statistical calculations. The difference among groups was settled with ANOVAs for normally distributed data or Kruskal-Wallis tests for skewed data, chi-square test was used for counting data; *Spearman’s* test was used to detect the correlation; logistic regression was used to analyze the risk factors for PAD; the optimum cutoff level was analyzed using the receiver operating characteristics (ROC) curve, and the area under the curve (AUC) was calculated. *p*<0.05 was considered statistically significant.

## 3 Results

### 3.1 Demographic and clinical characteristics

**Figure 1:**
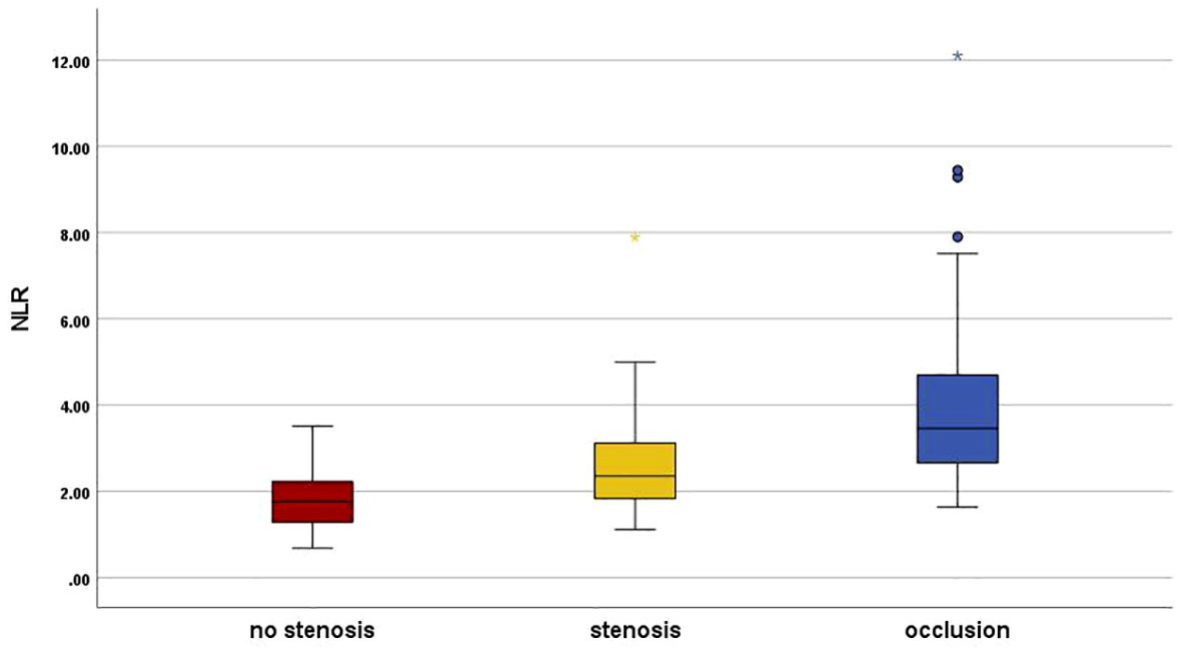
Neutrophil–to–lymphocyte ratio distribution in the study groups. Graphical representations of NLR distribution are shown by box plot. Group L1 (Red), plaque with no stenosis; group L2 (Yellow), plaque with luminal stenosis<99% and group L3 (Blue), total vascular occlusion in lower limb arterial. NLR, the neutrophil-to-lymphocyte ratio. *There was a statistically significant difference in post hoc analysis between the three groups

Patients were classified into group L1 (N=56, 28.9%), group L2 (N=58, 29.9%) and group L3 (N=80, 41.2%). The demographic statistics and clinical characteristics of the patients are presented in **Table 1**. No pronounced change was noted among groups regarding sex, smoking, eGFR, TC, and TG. As revealed, the age (58.61 ± 7.38 vs 64.66 ± 8.94 vs 68.33 ± 9.52, *p*<0.001), disease duration (7.5 vs 10.0 vs 10.0, *p*=0.01), BMI (22.2 vs 23.2 vs 25.6, *p*=0.032), and prevalence of hypertension (35.7% vs 65.5% vs 77.5%, *p*=0.002) were considerably higher in group L2 and group L3 than in group L1, systolic blood pressure was significantly higher in group L3 (127.5 vs 125.0 vs 150.0, *p*<0.001), but this change was not related to diastolic blood pressure (80.0 vs 77.0 vs 74.0, *p*=0.601); there was no notable difference in the WBC counts, while the neutrophil counts was higher in group L3 (3.30 vs 3.51 vs 4.30, *p*=0.012) and the lymphocyte counts was lower compared with group L1 (1.96 vs 1.68 vs 1.38, *p*=0.012); CRP was significantly higher in the group L3 than in group L1, but there was no significant difference between them and group L2 (1.69 vs 1.97 vs 2.26, *p*=0.036), HbA1c gradually increased in three groups (8.6 vs 8.9 vs 9.65, *p*=0.045); in the analysis of lipid profiles, there was a progressive increase in LDL-c (2.31 vs 2.52 vs 2.88, *p*=0.006) accompanied by a mild HDL decline (1.15 vs 1.11 vs 1.05, *p*=0.020), and this change was particularly significant in L3. In addition, with increasing stenosis confirmed by angiography, there was a progressive decrease in ABI (1.11 vs 1.02 vs 0.94, *p*<0.001) accompanied by an increase in NLR (1.76 vs 2.35 vs 3.60, *p*=0.001). Graphical representations of NLR distribution are shown by box plot in **Figure 1**.

### 3.2 Multivariable analysis on risk factors for PAD

The variables with *p*<0.05 among the above univariate factors (shown in **Table 1**) were included in the logistic regression model for multivariable analysis, we found that NLR, ABI, age, BMI, systolic blood pressure and LDL-c were independent influences on PAD (*p*<0.05, **Table 2**). Remarkably, ABI values were negatively correlated with the severity of PAD stenosis (OR=0.025, *p*=0.01), whereas all the other factors were facilitators (OR>1); compared with the mild changes in Neutrophil count and Lymphocyte count (*p*>0.05), the elevation of NLR was more pronounced (OR=2.136, *p*=0.001). Lastly, the disease duration, HbA1c, CRP and high-density lipoprotein cholesterol did not have any independent effect on the onset of PAD (*p*>0.05).

**Table 2:** The multivariable logistic regression analysis for factors associated with PAD.

Independent variables	*β*	SE	OR	95% CI	Wald	*p*-value
Age	0.077	0.027	1.080	1.024-1.138	8.288	***0.004***
BMI	0.235	0.112	1.265	1.015-1.578	4.369	***0.037***
SBP	0.042	0.020	1.043	1.003-1.083	4.566	***0.033***
LDL-c	1.043	0.424	2.838	1.236-6.514	6.050	***0.014***
NLR	0.759	0.226	2.136	1.370-3.327	11.255	***0.001***
ABI	-3.703	2.252	0.025	0.000-2.034	6.704	***0.010***

The variables with p<0.05 among the above univariate factors (shown in **Table 1**) were included in the logistic regression model for multivariable analysis.

BMI, body mass index; SBP, systolic blood pressure; LDL-c, low-density lipoprotein cholesterol; NLR, the neutrophil-to-lymphocyte ratio; ABI, ankle-brachial index. Bold values indicate statistical significance.

### 3.3 Correlation analysis on NLR and ABI in different groups

*Spearman’s* correlation analysis showed a negative correlation between ABI and the group, while the relationship between the NLR and group was positive and more relevant (*Rho*= -0.471, *p*<0.001; *Rho*=0.609, *p*<0.001, respectively. Shown in **Table 3**).

**Table 3:** The *Spearman’s* relationship between the Group and variables.

Variables		Duration	BMI	SBP	LDL-c	HbA1c	NLR	ABI
Group	*Rho*	0.298**	0.417**	0.511**	0.496**	0.309**	0.609**	-0.471**
	*p*	***0.006***	***<0.001***	***<0.001***	***<0.001***	***0.002***	***<0.001***	***<0.001***

**At the 0.01 level (two-tailed), the correlation was significant.

BMI, body mass index; SBP, systolic blood pressure; HbA1c, Hemoglobin A1c; LDL-c, low-density lipoprotein cholesterol; HbA1c, Hemoglobin A1c; NLR, the neutrophil-to-lymphocyte ratio; ABI, ankle-brachial index. Bold values indicate statistical significance.

### 3.4 Predictive role of elevated NLR and decreased ABI in severe PAD

**Figure 2:**
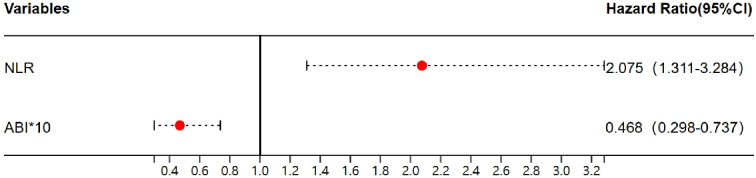
The forest plot of NLR and ABI in severe PAD. NLR, the neutrophil-to-lymphocyte ratio; ABI, ankle-brachial index.

**Figure 3:**
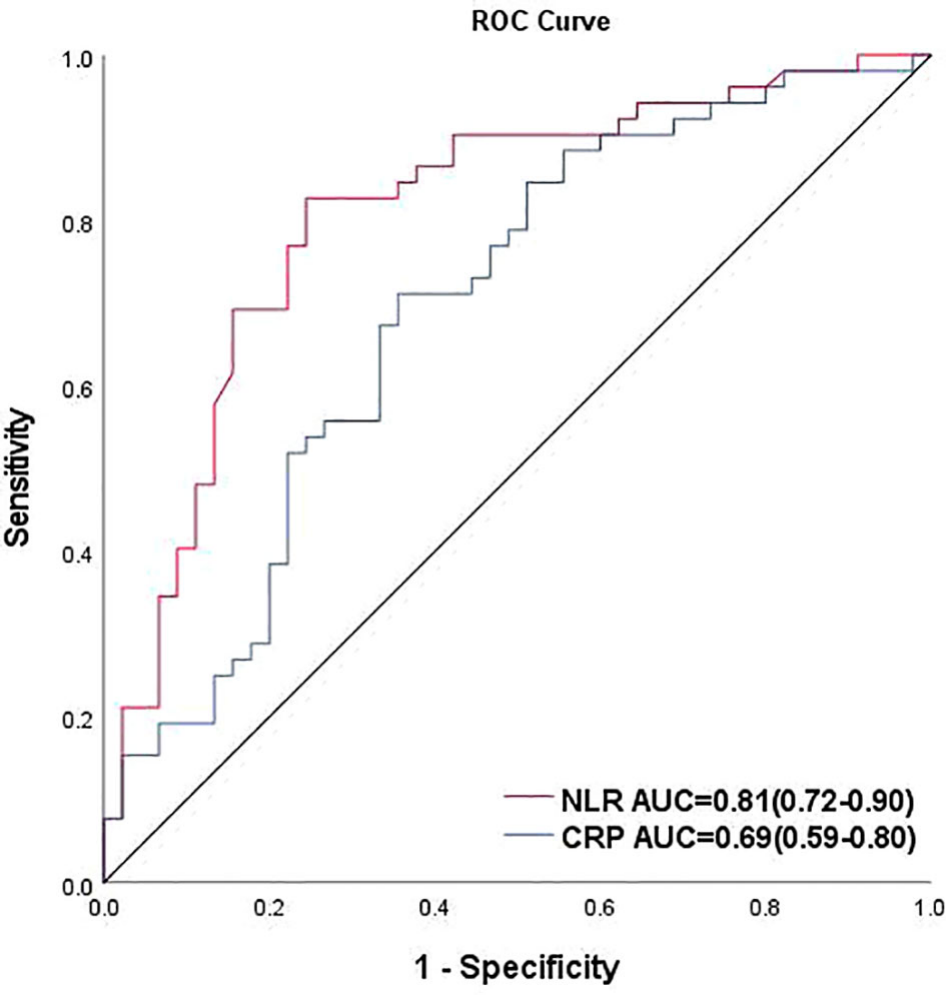
Receiver operating characteristic curve of NLR and CRP for predicting severe PAD. AUC, area under the curve; cutoff value: the maximum value of the Jordon index also corresponds to the optimal diagnostic threshold of the method.

Binary logistic regression analysis with the occurrence of severe stenosis (>70%) showed that the probability increased to 2.075 times for every 1 increase in NLR, while 46.8% for every 0.1 increase in the ABI (**Table 4**, **Figure 2**). Finally, to assess the NLR prediction performance compared with CRP, we constructed an ROC curve and calculated the AUC, showing that AUC of NLR was better than CRP at predicting severe stenosis(0.81 vs 0.69), meanwhile the NLR optimal cutoff value of 2.73 with a sensitivity of 82.7% and a specificity of 75.6%, whereas the cutoff point of 1.58 was taken to have a sensitivity of 100% and a specificity of 9.8% (**Figure 3**).

**Table 4:** Binary logistic regression analysis of NLR and ABI for predicting severe PAD.

	β	*p*-value	OR	95% confidence interval	
NLR	.730	***.002***	2.075	1.311	3.284
ABI*10	-.759	***.001***	.468	.298	.737

Severe PAD, stenosis >70% in PAD.

NLR, the neutrophil-to-lymphocyte ratio; ABI, ankle-brachial index; OR, Odds Ratio. p<0.05 is marked with bold font.

## 4 Discussion

Diabetic peripheral vascular disease is one of the most common complications in diabetic patients, which can lead to disability and amputation due to atherosclerotic occlusion of the lower limbs (4, 38), inflammatory factors and inflammatory cells play an important role in this, such as neutrophils and lymphocytes.

NLR is a novel marker of systemic inflammatory response that stably responds to the inflammatory state of the body. NLR intermixes the predicted risk of two leukocyte subtypes into a single factor, which has a stronger predictive value, and can minimize the effects of physiological conditions (dehydration, sample error) compared to changes in absolutes count or lymphocytes alone. Previous studies have indicated that NLR is elevated in patients with coronary arteriopathy, but the differences in absolute neutrophil and lymphocyte counts are not statistically significant, suggesting that NLR has its unique sensitivity in response to arteriopathy (39). In diabetic retinopathy and nephropathy, NLR also has an important role in predicting the severity of complications in patients (35, 36, 40, 41). In addition, NLR can predict the prognosis of heart failure (29, 42), cerebral infarction (43), pancreatitis (44, 45), and other acute and chronic inflammation-related diseases, and it is closely related to the severity of these diseases, which can be used as an evaluation index of clinical effect, thus becoming a point of special interest in recent years.

At present, no study has been found on the correlation between diabetic PAD and NLR, so this paper mainly investigates the relationship between the development of lower limb arteriopathy in diabetes mellitus and the change of NLR, results suggest that: NLR ratio was critically higher in the stenosis or occlusion group compared with the no-stenosis group, it is positively correlated with the degree of stenosis, accompanied by the progressive decline of the ABI, this conclusion is consistent with the findings of a meta-analysis demonstrated that high NLR values increased the risk of coronary artery disease (CAD) 1.62-fold and the risk of stroke 3.86-fold (46), Onofrei V also found that NLR was higher in patients with severe obstruction in PAD without diabetes (47), these studies confirm the important role of NLR in promoting the development of various types of peripheral arterial disease. Patients presenting with arterial stenosis and occlusion showed older age, longer disease duration, bigger body mass index, and higher prevalence of hypertension than those in the group without stenosis, as well as being at high risk for needing early intervention, showed the same trend as characteristics of the population with other vascular diseases (48–50), suggests that these influences may be common risk factors for various cardiovascular diseases. HbA1c was found to be progressively elevated with the aggravation of stenosis, confirming that poor glycemic control was a determinant of the severity of PAD, this finding was consistent with the studies by other researchers (33, 34, 51). In the lipid profile, LDL-c increased significantly in severe PAD while HDL tended to decrease gradually, a finding consistent with previous studies in other vascular diseases (52, 53). Surprisingly, TG and TC were not independent risk factors for the development of PAD, Bertrand C found the similar result, they described the independent associations between HDL-cholesterol, total cholesterol/HDL-cholesterol ratio or non-HDL-cholesterol and the prevalence of major PAD in people with type 2 diabetes, neither TG nor TC (54); more importantly, the fluctuations in TG were greater compared to changes in LDL and HDL therefore the differences between groups were smaller, a similar study by Gillian M Keating was also reported (55); another possible reason cannot be excluded is due to the fact that patients with higher blood glucose have higher use of lipid-regulating drugs (56), some participants were on statin therapy leaving some uncertainty during follow-up that may possibly bias our results. However, Kuo-Cheng Chang found no significant correlation between peripheral neuropathy in type 2 diabetes mellitus and lipid levels and statin usage (57). Lastly, the role of smoking does not seem to be as important as expected, which is not consistent with most other studies (58–60), however, similar findings were reported in the study by Al-Momany A on type 2 diabetic nephropathy and smoking (61), it’s not the only case, O’Donnell TFX (62) found that black patients were younger, less likely to smoke, but more likely to have diabetes, limb-threatening ischemia. As to our study, the specific reason for this may be related to the differences in the distribution of smokers by gender and the fact that some ex-smokers are currently quitting.

In order to accurately screen patients who truly need timely angiography and intervention, and to avoid the waste of medical resources caused by non-essential examination, we set diabetes mellitus combined with “severe stenosis” of the lower extremity vessels (>70%) as the critical intervention point to explore the cutoff value of NLR. Binary Logistic regression analysis showed an important role of imbalance in the ratio between neutrophils and lymphocytes in lower extremity arterial stenosis as a positive predictor. Previous studies have shown that the development of atherosclerotic disease is positively associated with the inflammatory factors CRP and IL-6 (63–65), which revealed the role of traditional biomarkers in risk assessment and highlights the strong association between inflammation and CVD. In our study, we compared the ROC curves of CRP and NLR, found that CRP (AUC: 0.69, 95%CI: 0.59-0.80) was worse than NLR (AUC: 0.81, 95%CI: 0.72-0.90) at predicting severe PAD. Coincidentally, Hoes LLF et al. recently published a study showing that NLR is the inflammatory marker that is more strongly related to CVD risk than CRP in patients with T2D (66), while Huang L also found that the diagnostic value of NLR was better than CRP in patients with the anti-synthetase sydrome (67). The Edinburgh Artery Study compared IL-6 with the major inflammatory marker CRP and found that IL-6 was an earlier predictor of worsening ABI values at 12 years of follow-up (68). In predicting coronary artery disease (69), the mean AUC for IL-6 and CRP were 0.74 (95% CI: 0.57-0.84) and 0.60 (95% CI: 0.44-0.74), respectively. In our study, elevated NLR predicted the onset and severity of PAD, which is consistent with the role of traditional inflammatory factors, what’ more, it can be hypothesized that NLR has a higher predictive power than traditional factors.

By ROC curve analysis, it was found that NLR plays a high predictive role in determining whether the stenosis of PAD is severe or worse, Arbel et al. (70) demonstrated that an NLR value above three is associated with a relative risk of 2.45 regarding the existence of sub-occlusive coronary lesions. In our study, taking the cutoff point of 1.58, it has 100% sensitivity and 9.8% specificity, while the optimal cutoff point of 2.73 appeared to have the maximum Youden index and the highest prediction efficiency. That is to say, if the NLR is less than 1.58, the lower extremity arterial ultrasound examination combined with ABI examination can be firstly used to evaluate the lower extremity vascular condition, there is no need to prefer digital imaging, while if the NLR is more than 2.73, DSA imaging or even interventional treatment is strongly recommended.

Currently, there are studies on NLR in diabetes mellitus combined with other peripheral vascular diseases such as CVD, stroke, NLR in patients with PAD without diabetes mellitus, but there are no studies focusing on the changes in NLR in diabetes mellitus combined with PAD, so this study fills this gap and find a more cost-effective way of screening patients with diabetes mellitus with PAD from a novel perspective. However, this research has some imperfections. Firstly, the research was only carried out in one single center, and the sample size was small. Secondly, due to the retrospective design of the study, many factors could not be involved. Lastly, as neutrophil and lymphocyte numbers may alter over time, it is a changing marker. Hence, further investigations are still required with longer follow-up, while larger samples are need to confirm their effectiveness as probable risk factors for PAD.

## 5 Conclusion

In our study, we demonstrated the predictive value of the NLR and its importance in the evaluation of patients with severe obstruction of the PAD. Indeed, NLR is derived from blood routine, simple and easy to obtain and perform in patients with suspected PAD, the use of ABI in combination with NLR may help to better identify patients with severe lower extremity arterial stenosis earlier, more conveniently, and noninvasively. This change in NLR makes it possible to become a new indicator for assessing the severity of lower-extremity peripheral arterial disease.

It is hoped that, in the future, there will be greater interest in and further studies on this topic to provide stronger recommendations to clinicians for an earlier diagnosis of the disease.

## Data Availability

The original contributions presented in the study are included in the article/supplementary material. Further inquiries can be directed to the corresponding authors.
